# Biosynthetic potential of uncultured Antarctic soil bacteria revealed through long-read metagenomic sequencing

**DOI:** 10.1038/s41396-021-01052-3

**Published:** 2021-07-12

**Authors:** Valentin Waschulin, Chiara Borsetto, Robert James, Kevin K. Newsham, Stefano Donadio, Christophe Corre, Elizabeth Wellington

**Affiliations:** 1 School of Life Sciences, University of Warwick, Coventry, UK; 2 Quadram Institute, Norwich, UK; 3 NERC British Antarctic Survey, Cambridge, UK; 4 NAICONS Srl, Milano, Italy; 5 Department of Chemistry, University of Warwick, Coventry, UK

**Keywords:** Metagenomics, Antibiotics, Microbial ecology, DNA sequencing

## Abstract

The growing problem of antibiotic resistance has led to the exploration of uncultured bacteria as potential sources of new antimicrobials. PCR amplicon analyses and short-read sequencing studies of samples from different environments have reported evidence of high biosynthetic gene cluster (BGC) diversity in metagenomes, indicating their potential for producing novel and useful compounds. However, recovering full-length BGC sequences from uncultivated bacteria remains a challenge due to the technological restraints of short-read sequencing, thus making assessment of BGC diversity difficult. Here, long-read sequencing and genome mining were used to recover >1400 mostly full-length BGCs that demonstrate the rich diversity of BGCs from uncultivated lineages present in soil from Mars Oasis, Antarctica. A large number of highly divergent BGCs were not only found in the phyla Acidobacteriota, Verrucomicrobiota and Gemmatimonadota but also in the actinobacterial classes Acidimicrobiia and Thermoleophilia and the gammaproteobacterial order UBA7966. The latter furthermore contained a potential novel family of RiPPs. Our findings underline the biosynthetic potential of underexplored phyla as well as unexplored lineages within seemingly well-studied producer phyla. They also showcase long-read metagenomic sequencing as a promising way to access the untapped genetic reservoir of specialised metabolite gene clusters of the uncultured majority of microbes.

## Introduction

Throughout the last century, bacterial natural products have proven invaluable for humankind. Their diversity has been harnessed to treat different ailments, and above all, to fight infectious disease. However, their biological roles and even the extent of their diversity are not well understood. Over the last decade, metagenomics has shown that a vast amount of the bacterial diversity on Earth is comprised of uncultured bacterial taxa, with 97.9% of bacterial operational taxonomic units estimated as unsequenced [1]. First efforts to characterise and harness the specialised metabolite diversity encoded in metagenomes have shown promising results [2–4]. Metagenomic library screenings have yielded novel compounds, among them antibiotics [3, 5, 6], while sequence-based studies have documented their diversity. In a study of grasslands with 1.3 Tb of short-read sequence data, Crits-Christoph et al. recovered hundreds of metagenome-assembled genomes (MAGs) obtained through a combination of binning approaches [7]. Analysis of the MAGs revealed a large number of biosynthetic gene clusters (BGCs) in Acidobacteria and Verrucomicrobia, widespread but underexplored phyla of soil bacteria. Analysis of nonribosomal peptide synthetase (NRPS) and polyketide synthase (PKS) domains indicated that NRPS and PKS from these groups were highly divergent from known BGCs of these classes. Borsetto et al. also reported a high degree of diversity of NRPS and PKS domains in Verrucomicrobia and other difficult-to-culture phyla [8]. Finding efficient ways to access this treasure trove of diverse and unexplored specialised metabolites will expand our understanding of microbial natural products, yield novel and useful compounds, and be an important step towards the development of much-needed antimicrobials.

Recent advances in long-read sequencing technology have made it possible to recover largely complete genomes metagenomic sequencing projects. A sequencing effort of 26 Gb returned 20 circular genomes from human stool samples [9], while a recent study using 1 Tb of long-read data from wastewater treatment plants recovered thousands of high-quality MAGs, 50 of which were circular [10]. Using mock community data, Pérez et al. demonstrated that full-sized BGCs could be successfully recovered from long-read metagenomic sequencing [11]. In light of recent advances in PCR-based cloning techniques that comprise heterologous expression of BGCs based on PCR amplification [12–15], the recovery of full-length metagenomic BGC sequences is promising as these sequences would be amenable to PCR amplification.

In recent years, a number of tools to explore and understand BGC diversity have been developed. Genomes can be mined for known classes of BGCs using tools such as antiSMASH [16], while the MIBiG database [17] links BGCs to known compounds. BGCs can be compared in networking-based tools such as BiG-SCAPE [18] and BiG-SLiCE [19] to assess relations of BGCs and estimate their novelty relative to extant BGC databases.

The isolated, harsh and unique environments of Antarctica show high degrees of endemism in their bacterial life, but their diversity remains underexplored [20]. Little is known about the specialised metabolites of Antarctic microorganisms. Few studies have explored polar, and specifically Antarctic, natural products using functional screening of isolates and metabolomics [21–25]. A high number pigmented bacterial isolates indicates that carotenoids and PKS, among other pigments, could be abundant BGC classes [26]. One culturing study suggested that Antarctic isolates show a below average potential for antimicrobials [21]. On the other hand, a primer-based study showed a promising diversity of NRPS and PKS diversity in soil from Mars Oasis in the southern maritime Antarctic [8], a site with exceptionally high diversity of micro- and macroorganismal life for its latitude [27, 28]. Low-temperature, aerated Antarctic soils have previously also been linked to methanotrophy [29, 30], and these soils could therefore harbour methanobactins, small ribosomally synthetised peptides that scavenge copper needed for methane monooxygenases.

In the present study, we used long-read shotgun metagenomic sequencing coupled with genome mining and bin- and contig-based taxonomic classification to analyse the biosynthetic potential of soil from Mars Oasis. We recovered >1400 highly diverse and mostly full-length BGCs from largely uncultured and underexplored bacterial phyla such as Acidobacteriota, Verrucomicrobiota and Gemmatimonadota as well as hitherto uncultured members of Proteobacteria and Actinobacteriota. This helps elucidate the biosynthetic diversity and highlights potential applications of the underexplored Antarctic soil microbiome. The present study further demonstrates how long reads make BGC recovery, analysis and taxonomic classification from highly complex metagenomes feasible even at low sequencing efforts (72.4 Gb).

## Materials and methods

### Site description

**Fig. 1:**
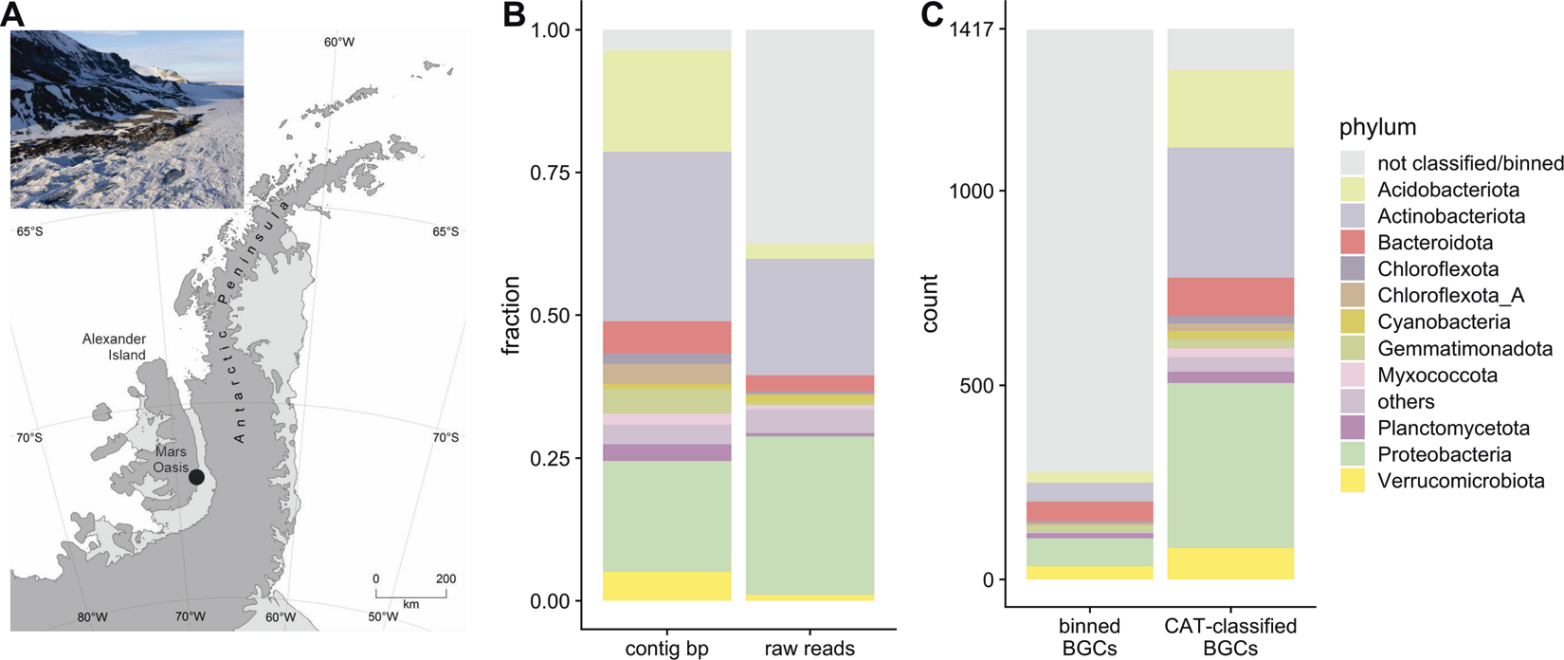
Sample location and phylogenetic classification of contigs, reads and BGCs. **A** Map of the Antarctic Peninsula with Mars Oasis indicated. Inset: Aerial photo of the site taken in austral summer. **B** Phylogenetic classification of contigs (by CAT) and long reads (by kraken2). **C** Phylogenetic classification of BGC-containing contigs using binning and CAT classification approaches.

Mars Oasis is situated on the south-eastern coast of Alexander Island in the southern maritime Antarctic at 71° 52′ 42″ S, 68° 15′ 00″ W (Fig. 1A). Mean soil pH is 7.9, with NO_3_^−^-N and NH_4_^+^-N concentrations of 0.007 and 0.095 mg kg^−1^, and total organic C, N, phosphorus and potassium concentrations of 0.26%, 0.02%, 8.01% and 0.22%, respectively. Soil moisture concentrations range between 2 and 6% in December–February, when snow or rainfall events are very rare, with the majority of precipitation falling as snow between March and November. Mars Oasis has a continental Antarctic climate, with frequent periods of cloudless skies during summer, when temperatures at soil surfaces reach 19 °C. During midwinter, the temperatures of surface soils decline to −32 °C. Mean annual air temperature is *c*. −10 °C [31].

### Soil sample, extraction and sequencing

One sample of surface soil (*c*. 0–50 mm, *c*. 2.5 kg) was collected with clean spades from the lower terrace at Mars Oasis (S71 52.691, W68 14.943) by British Antarctic Survey staff on December 8, 2017 and was kept cool for several hours before being stored at −20 °C. Soil was kept at this temperature until being thawed for DNA extraction. A gentle chemical lysis and DNA extraction of 50 g of soil were performed and the DNA was subjected to size selection to approximately 20 Kb and larger by agarose gel electrophoresis using a protocol previously used for metagenomic library construction [32]. DNA was sequenced using Oxford Nanopore Technologies (ONT) MinION and Illumina HiSeq 150 bp paired-end reads. For long reads, the DNA was sequenced using three R9.4.1 flow cells and the SQK-LSK109 kit. The nuclease flush protocol was used between each independent library run on a flow cell. Short-read DNA library preparation and Illumina sequencing were performed by Novogene according to their in-house pipeline. In short, 1 µg of DNA was sheared to 350 bp, then prepared for sequencing using NEBNext DNA Library Prep Kit. The library was enriched by PCR and underwent SPRI-bead purification prior to sequencing on a HiSeq sequencing platform.

### Read processing, assembly, polishing and quality control

The long reads fast5 data were basecalled with Guppy v.3.03 (HAC model). Basecalled raw reads were assembled using Flye v2.5 using the -meta flag. The resulting assembly was polished with four iterations of Racon v1.4.7 [33] followed by one run of Medaka v0.7.1 [34]. Then, the short reads were used for six rounds of polishing with Pilon v1.23 [35]. The approximate assembly quality was checked at every step using Ideel [36]. Long reads were also classified with kraken2 2.0.7b using the Genome Taxonomy Database (GTDB) r89 database. Short reads were used to estimate diversity and predict coverage with nonpareil 3.304. Furthermore, short reads were assembled with SPAdes 3.14.1 using the -bio flag (“biosyntheticSPAdes”). Read and assembly statistics can be found in the “Results” section (Table 1). Initial assessment of potential indels showed that 82% of all proteins were shorter than 0.9 times the length of the closest reference protein in the UniProt database and 7.2% were longer than 1.1 times the length of the closest reference protein. After polishing using Racon, Medaka and Pilon, the proportion of potentially truncated proteins was reduced to 70%, while that of proteins that were potentially too long slightly increased to 7.6%.

**Table 1:** Raw sequence, polished assembly, BGC mining and binning statistics.

Nanopore reads	No. of reads	9.3 million
	Total length	44.4 Gb
	N50	9.4 Kb
150 bp PE Illumina reads	No. of reads	186.6 million
	Total length	28 Gb
Nonpareil analysis	Abundance-weighted coverage at 44.4 Gb	85.3%
	Diversity *N*_*d*_	21.6
Polished assembly	No. of contigs	48422
	Length	2.4 Gb
	N50	84.8 Kb
	Max length	129.6 Kb
antiSMASH BGCs	No. of BGCs	1417
	BGCs on contig edge	564
	Total length	40.5 Mb
	Mean length	28.5 Kb
	Max length	129.6 Kb
metaWRAP 50/10 bins	No. of bins	114
	Mean no. of contigs per bin	18.5
	BGCs in bins	278
	Average bin N50	224 Kb

### Genome mining, binning, taxonomic assignment and quality control

For detecting BGCs, the polished assembly was analysed by antiSMASH v5.1 [16]. For taxonomic assignment of contigs, proteins were predicted using Prodigal [37], and CAT [38] (settings -sensitive -r 10 and -f 0.3) was used with a DIAMOND [39] database built from proteins in the GTDB_r89_54k database [40] as well as the NCBI non-redundant protein database. The contigs were also binned with MetaBAT2 [41], CONCOCT [42] and MaxBin2 [43], using long- and short-read abundance profiles generated with bowtie2 [44] and minimap2 [45] as a proxy for differential coverage. The resulting bins were subjected to metawrap-refine [46] to produce the final bins and classified using GTDB-Tk 0.3.2 (r89). BiG-SCAPE [18] 1.0.1 was run in -auto mode with -mibig enabled to identify BGCs families. Networks using similarity thresholds of 0.1, 0.3, 0.5 and 0.7 were examined, since higher thresholds led to extensively large proposed BGC families. In order to calculate BGC novelty, BiG-SLiCE 1.1.0 [19] was run in -query mode with a previously prepared dataset which had been computed from 1.2 million BGCs using -complete_only and *t* = 900 as threshold [47]. The resulting distance *d* indicates how closely a given BGC is related to previously computed gene cluster families (GCFs), with a higher *d* indicating higher novelty. For this analysis, we highlighted values of *d* > t and *d* > 2t (i.e. *d* > 900 and *d* > 1800, respectively), as they were previously suggested as arbitrary cutoffs for “core”, “putative” and “orphan” BGCs [47].

### Precursor peptide homology searches and sequence logo construction

ORFs were aligned using Clustal Omega [48] and a HMMER [49] search was performed in the EBI reference proteome database with a cut-off *E*-value of 1E−10. The resulting protein sequences were aligned using Clustal Omega and a HMM was generated and visualised using skylign.org [50].

## Results

### Soil diversity, taxonomic classification and binning of BGCs

Nonpareil analysis estimated an abundance-weighted coverage of 85.3% for the 44.4 Gb used in the long-read assembly. To achieve 95% and 99% coverage, respectively, 250 Gb and 1.6 Tb of sequencing were predicted to be necessary. Alpha diversity was estimated at *N*_*d*_ = 21.6. Contigs were binned using CONCOCT, MaxBin2 and MetaBAT2, consensus bins were generated using metaWRAP refine and classified using GTDB-Tk. This yielded 114 bacterial bins with CheckM completeness > 50% and contamination < 10% containing 278 BGCs (see Table 1.) Since only 278 BGCs had been binned, an additional contig-based classification approach was adopted. All contigs were classified using CAT with a database based on GTDB r89 proteins, leading to a classification of 93% of BGC-containing contigs at a phylum level (Fig. 1B, C). A cross-check of bin-level classification and contig-level classification of the 269 binned and CAT-classified BGC-containing contigs showed three conflicts at different levels in total (phylum: 0, class: 1, order: 1, family: 0, genus: 1, species: 0). Of the 2892 total binned and CAT-classified contigs, 52 (1.7%) were classified differently at order level using CAT. This indicates that the risk of misclassification of BGC-containing contigs by CAT is low, but cannot be excluded. Bin-level classification was preferred where available.

### Recovery of diverse and full-length BGCs

The polished assembly was analysed using antiSMASH v5.1. A total of 1417 BGCs were identified on 1350 contigs (Table 1). A total of 564 BGCs (39.8%) were identified as being on a contig edge and were therefore categorised potentially incomplete, while 853 (60.2%) were full length. The most abundant classes of BGCs were terpenes (27.2%), followed by NRPS (15.7%) and bacteriocins (10.1%). In particular, terpenes were dominated by few subclasses. Out of 401 observed terpene BGCs, 321 contained a squalene/phytoene synthase Pfam domain (PF00494). This indicates that the product of these BGCs is a tri- or tetraterpene. Forty-four BGCs also contained a squalene/hopene cyclase (N terminal; PF13249), 39 BGCs contained a carotenoid synthase (PF04240), while 47 contained a lycopene cyclase domain (PF05834).

Approximately half of the ribosomally synthesised and post-translationally modified peptides (RiPPs) identified in the sample contained methanobactin-like DUF692 domains (PF05114). However, no BGCs resembling known methanobactin BGCs were found.

The proportion of proteins identified as too short on BGC-containing contigs was estimated at 63%. It is possible that this measure was influenced by the UniProt reference database not containing representative proteins for the mostly uncultivated strains recovered in this study. However, fragmentation of ORFs through indels was clearly visible, especially in NRPS and PKS BGCs in which whole megasynthase genes were broken up into several fragments.

### Long reads and GTDB improve phylogenetic classification of environmental BGCs

The use of GTDB proteins instead of the NCBI non-redundant protein database increased the classification success of BGC-containing contigs from 36.8% classified at order level with the NCBI database to 71.8% with GTDB. The difference was mainly due to BGCs from MAG-derived orders which were not present in the NCBI database, such as UBA7966. However, the GTDB database is also much smaller than the NCBI nr database, and many MAG-derived clades especially at lower taxonomic ranks do not have many representatives in the GTDB database. To avoid misclassifications, we therefore decided to conduct analysis at class and order level, even if contigs were classified at lower taxonomic ranks.

To assess the advantages of long-read sequencing for BGCs detection and classification, the output was compared with BiosyntheticSPAdes, which allows the assembly of NRPS and PKS from short-read sequences by following an ambiguous assembly graph using a priori information about their modularity. Using BiosyntheticSPAdes with the 28 Gb of short reads, 228 unambiguous NRPS/PKS BGCs were predicted. Sixty one of these were above 5 Kb long and five NRPS were larger than 30 Kb. Furthermore, 202 other BGCs were predicted from other contigs. 96.7% of BGCs were marked as on a contig edge, i.e. not full length. Indeed, 392 out of 430 BiosyntheticSPAdes BGCs could be aligned to 255 long-read BGCs using blastn (*E*-value < 1E−90), indicating that mostly the same BGCs were assembled, but they were fragmented in the short-read assembly (see Supplementary Fig. 1). In the case of NRPS/PKS BGCs, even the BGCs on contigs with the highest coverage (>120×) were fragmented into two or more contigs. Classification success using the same binning and CAT approach was lower (68% at phylum level, 50% at order level; 48 BGCs binned). This could be attributed to the lack of genomic context around the BGCs. While BiosyntheticSPAdes predicted a large number of BGCs in total, the practical usability and interpretability of the output remained low, since completeness, cluster borders and potential modification genes could not be assessed and phylogenetic classification success was reduced.

### Highly divergent BGCs found in unusual specialised metabolite producer phyla

**Fig. 2:**
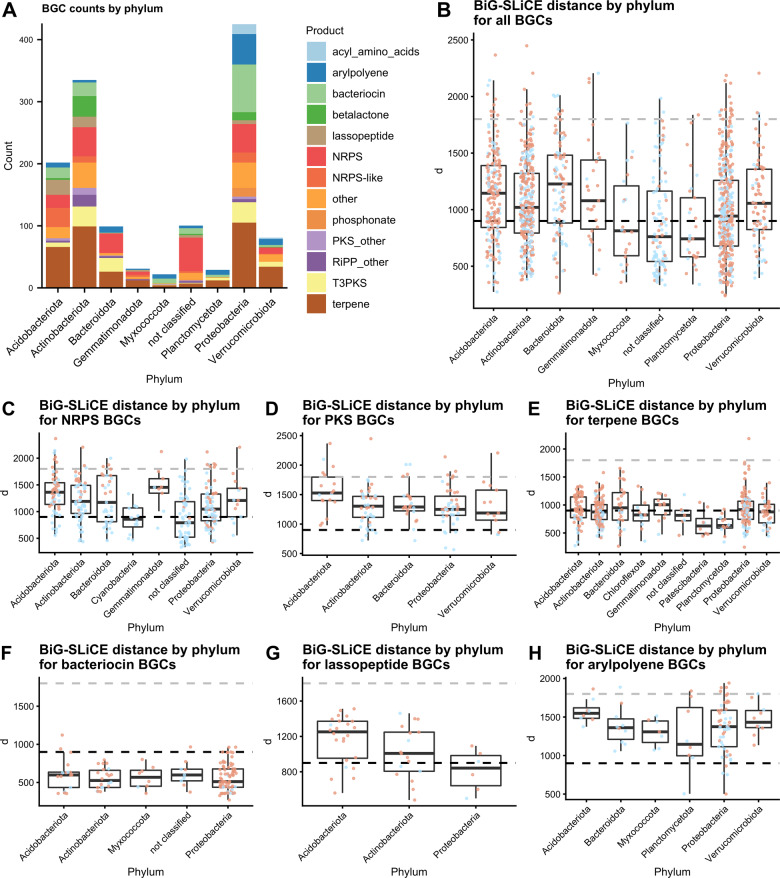
Phylum-level BGC distribution and BiG-SLiCE distances. **A** BGCs by phylum and BGC type (phyla with a count < 20 removed; products with count < 10 under “others”. **B** BiG-SLiCE distances of BGCs by phylum, with the black dotted line indicating *d* = 900 and the grey dotted line *d* = 1800 (phyla with a count < 20 removed). **C**–**H** BiG-SLiCE distances for different BGC types plotted by phylum (phyla with <5 BGCs of the type removed; hybrid BGCs counted for both classes). Each point indicates a BGC. Salmon = BGC not on contig edge, Light blue = BGC on contig edge.

Examination of the BGC counts by BGC type and phylum showed that the three well-known producer phyla Actinobacteriota, Proteobacteria and Bacteroidota together contributed over 60% of BGCs (Fig. 2A). BGCs attributed to Acidobacteriota and Verrucomicrobiota represented up to 20% of the total BGCs, while other phyla constituted the remaining 12%, and 7% remained unclassified at phylum level. In particular, 20% of NRPS remained unclassified at phylum level. No archaeal BGCs were found.

The 1417 BGCs were then analysed with BiG-SLiCE’s query mode in order to calculate their distance (*d*) from a set of pre-computed GCFs comprised of 1.2 mio known BGCs. The analysis showed that 845 out of 1417 BGCs (59.6%) had a *d* > 900, indicating that they were only distantly related to a GCF. Fifty-five outliers were found with *d* > 1800, indicating extremely divergent BGCs. A wide span of distances was present within each phylum which indicates that each phylum contained BGCs that are both closely and distantly related to known BGCs (Fig. 2B). The median distances showed significant variation between phyla, with Bacteroidota containing the highest novelty (median *d* = 1227) and Planctomycetota the lowest (median *d* = 742). This overall score was, however, influenced by the fact that different classes of BGC scored differently. For example, NRPS/PKS BGCs scored higher than, e.g. terpenes or bacteriocins. Rankings of single BGC classes showed that the high Bacteroidota score was partly driven by the large number of NRPS (Fig. 2C) and the small number of terpenes and bacteriocins (Fig. 2E, F) in the phylum. This is evidenced by the fact that other phyla scored the highest in individual BGC classes. For NRPS BGCs, Gemmatimonadota, Acidobacteriota and Verrucomicrobiota showed the highest values for *d* (Fig. 2C). Gemmatimonadota furthermore showed the highest value for *d* when considering terpene BGCs (Fig. 2E), while Acidobacteriota scored high for lassopeptides, arylpolyenes and PKS (Fig. 2G, H, D). Furthermore, BGCs on a contig edge tended to score lower. To check whether low coverage and the resulting insertion and deletion errors in the assembly led to overestimation of *d*, contig coverage as well as percentage of correctly sized ORFs (as calculated by ideel) were plotted against *d*. There was a positive correlation of *d* values with increased coverage up until a coverage of ca 10, indicating an underestimation of novelty at low coverage. Similarly, for contigs with under 20% correctly sized ORFs, there was a slight positive correlation between the percentage of correctly sized ORFs and distance. As expected, coverage showed a strong positive correlation with percentage of correctly sized ORFs (see Supplementary Figs. 2–4).

### Acidobacterial BGCs

**Fig. 3:**
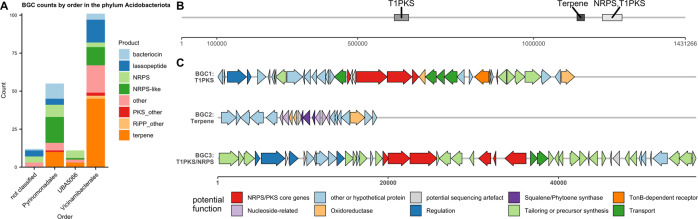
Order-level distribution of acidobacterial BGCs and BGC map of an acidobacterial contig. **A** BGC counts by BGC type and order in phylum Acidobacteriota. **B** Map of a large Acidobacteriota contig (order ﻿Vicinamibacterales) and the BGCs on it. **C** Cluster map of proposed functions of genes in BGC1, BGC2 and BGC3. Functions were predicted from BLASTing against NCBI nr database as well as antiSMASH module predictions. A detailed table of homologous proteins can be found in Supplementary files.

Analysis of acidobacterial BGCs by order (Fig. 3A) showed that terpenes were the most numerous, but with significant contributions from PKS, NRPS, lassopeptide and bacteriocin clusters. The orders of Pyrinomonadales and Vicinamibacterales constituted >60% of BGCs.

BiG-SCAPE analysis showed that BGCs mainly clustered together within orders (Supplementary Table 1). None of the families contained MIBiG clusters at the cutoffs used. Acidobacteriota showed a large number of lassopeptides, 16 of which grouped into two GCFs. NRPS-like BGCs also contributed a large number to the sample. In particular, one NRPS-like family from the order Vicinamibacterales showed homology to the VEPE BGC from *Myxococcus xanthus* (MIBiG BGC0000871). Furthermore, seven NRPS/PKS with a megasynthase gene length of over 20 Kb were found with the largest BGC measuring 89 Kb of NRPS and PKS megasynthase genes. The largest Acidobacteriota (order Vicinamibacterales) contig was 1.5 Mb in size and contained three BGCs: a PKS, a terpene and a NRPS/PKS hybrid cluster (Fig. 3B, C). BGC1 (*d* = 1397) contained a partial one-module NRPS followed by a partial PKS module as well as transporter genes and a TonB-dependent receptor protein, suggesting a role as a siderophore. BGC2 (*d* = 1103) contained squalene/phytoene synthase genes and several potential tailoring enzymes. BGC3 (*d* = 1977) contained a complete NRPS and a partial NRPS module and an incomplete PKS domains. Several gaps visible in the BGC make a sequencing error seem possible, leading to truncated genes and therefore missing domains.

### Verrucomicrobial BGCs

**Fig. 4:**
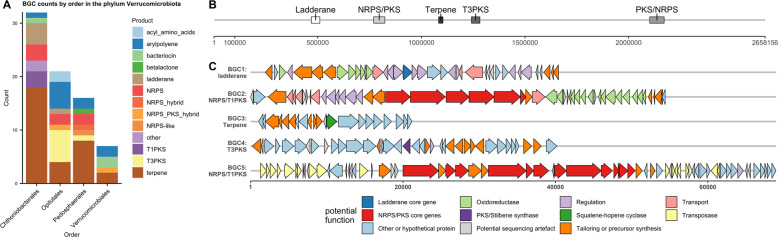
Order-level distribution of verrucomicrobial BGCs and BGC map of a verrucomicrobial contig. **A** BGC counts by BGC type and order in phylum Verrucomicrobiota. **B** Map of a large Verrucomicrobiota contig (order Opitutales) and the BGCs on it. **C** Cluster map of proposed functions of genes in BGC1–BGC5. Functions were predicted from BLASTing against NCBI nr database as well as antiSMASH module predictions. *X* axis represents basepairs. A detailed table of homologous proteins can be found in Supplementary files.

The analysis of Verrucomicrobial BGCs by order (Fig. 4A) showed that the vast majority of BGCs were terpenes, followed by arylpolyenes, PKS, NRPS, as well as ladderanes. The most prolific producer orders were Opitutales, Pedosphaerales and Chtoniobacterales. Verrucomicrobial BGCs did not show strong clustering into conserved GCFs compared to Acidobacteriota (Supplementary Table 2). One NRPS and one PKS BGC were the only BGCs that clustered with MIBiG clusters. The largest Verrucomicrobiota contig (order Opitutales) was 2.6 Mb in size and featured five BGCs, two of which were NRPS-PKS hybrids with megasynthase genes above 20 Kb (Fig. 4B, C). BGC1 (*d* = 1479) contained a ladderane-type 3-oxoacyl-[acyl-carrier-protein] synthase. BGC2 (*d* = 1305) contained four NRPS modules interspersed by one PKS module. BGC3 (*d* = 673) contained a squalene-hopene cyclase, indicating a role in hopanoid biosynthesis. BGC4 (*d* = 1142) encoded a chalcone/stilbene synthase. BGC5 (*d* = 1340) contained a PKS module followed by five NRPS modules. The third module, however, showed a truncated A domain, with the antiSMASH HMM NRPS-A_a3 only matching around 50 bp at the end of ORF ctg423_1968. This could be explained by a sequencing error in which an indel lead to a frameshift, causing a premature stop codon. Indeed, nucleotide-level BLAST of the gap between ctg423_1968 and the PCP-domain containing ctg423_1970 showed a match to known A domains. It is, however, not possible to rule out potential pseudogenisation.

### Uncultivated and underexplored classes and orders from Actinobacteriota and Proteobacteria show a large biosynthetic potential

#### Actinobacteriota: Acidimicrobiia and Thermoleophilia

**Fig. 5:**
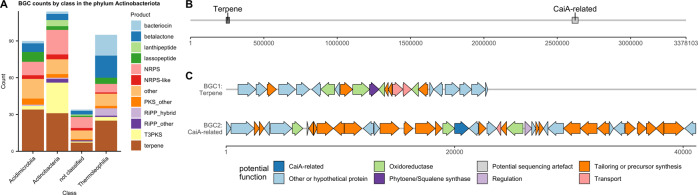
Class-level distribution of actinobacterial BGCs and BGC map of an actinobacterial contig. **A** BGC counts by BGC type and class in Actinobacteriota. **B** Map of a large Actinobacteriota contig (order IMCC26256) and number of basepairs. **C** Cluster map of proposed functions of genes in BGC1 and BGC2. Functions were predicted from BLASTing against NCBI nr database as well as antiSMASH module predictions. *X* axis represents basepairs. A detailed table of homologous proteins can be found in Supplementary files.

The phylum Actinobacteriota (335 BGCs) featured a large amount of BGCs unclassified at order level. Therefore, they were analysed by class (Fig. 5A). The class Actinobacteria (114 BGCs) contained BGC-rich genera such as *Streptomyces* and *Pseudonocardia* and accordingly contributed a large amount of BGCs in the sample. The class Acidimicrobiia (90 BGCs) contained the genera *Illumatobacter* and *Microthrix* and several uncultured genera. The class Thermoleophilia (95 BGCs) contained genera such as *Solirubrobacter* and *Patulibacter*, besides uncultured genera, and contributed to a large amount of the bacteriocin and betalactone BGCs. The amount of BGCs in these classes that were not placed into lower taxonomic ranks indicated that there is a large unexplored diversity of uncultured Actinobacteriota containing a great diversity of BGCs.

Remarkably, one circular genome from the uncultured order IMCC26256 from the class Acidimicrobiia was recovered in a single contig, measuring 3.3 Mb in size and containing two BGCs (Fig. 5B, C). The terpene BGC (*d* = 1398) contained a squalene synthase, a lycopene cyclase and polyprenyl synthetases, suggesting a role in pigment formation. The CaiA-related BGC (*d* = 1869) contained an acyl-CoA dehydrogenase related to CaiA (involved in saccharide antibiotic BGCs). BLAST hits indicated other genes related to small organic acids, sugars and nucleoside metabolism.

Two families of terpenes containing terpene cylases, methyltransferases and/or P450s showing similarity to the known geosmin and 2-methylisoborneol BGCs were found, with members belonging to both Acidimicrobiia, Thermoleophilia and unclassified Actinobacteriota. One BGC from a *Streptomyces* spp. was detected, containing an LmbU-like gene on the very edge of the contig. BiG-SCAPE analysis showed that Actinobacteriota BGCs mostly grouped within the classes, and one lanthipeptide BGC grouped with MIBiG BGCs at the cut-off used (Supplementary Table 3).

#### Proteobacteria: the uncultured methanotrophic order UBA7966 as a specialised metabolite producer

**Fig. 6:**
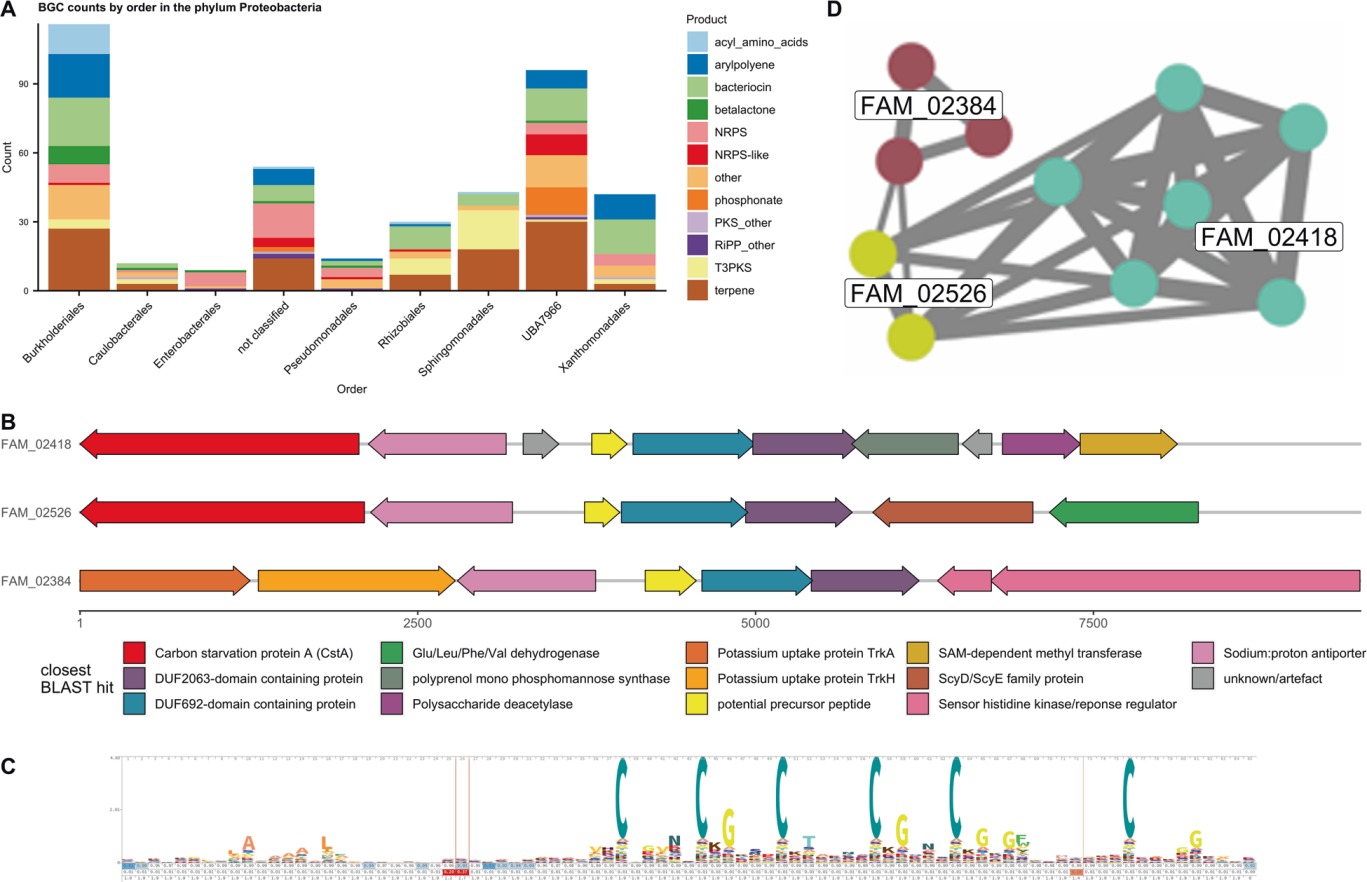
Order-level distribution of proteobacterial BGCs, BGC maps and analysis of DUF692 BGCs from order UBA7966. **A** BGC counts by BGC type and order in the phylum Proteobacteria. **B** Cluster layout of three gammaproteobacterial DUF692-containing BGCs representatives: contig_12391 for FAM_02418, contig_14956 for FAM_02526 and scaffold_15362 for FAM_02384. **C** Sequence logo generated from an HMM of 301 potential precursor peptides. **D** Similarity network generated from BiG-SCAPE with brown: FAM_02384, turquoise: FAM_02418, green: FAM_02526.

Analysis at the order level of proteobacterial BGCs showed that the biggest contributor was the Burkholderiales order with 116 BGCs (Fig. 6A) followed by order UBA7966 with 96 BGCs. UBA7966 BGCs included a variety of BGCs, including terpenes, bacteriocins, phosphonates, NRPS and NRPS hybrids, NRPS-like and arylpolyenes. In particular, the high abundance of NRPS-like and phosphonate BGCs in UBA7966 contrasted with the lower counts in other proteobacterial orders in the dataset. By order, UBA7966 contigs also showed a high average coverage of 26×, compared to the total average of 10.2×, indicating a high abundance. The total length of UBA7966 contigs was 53 Mb, indicating the presence of several genomes.

The order UBA7966 is an uncultured order consisting of one family, UBA7966, which contains two genera, *UBA7966* and *USCγ-Taylor*. UBA7966-family bin bin.3 was assigned no genus, while all CAT-assigned contigs were assigned species *USCγ-Taylor sp002007425*, the only species in the *USCγ-Taylor* genus. The *USCγ-Taylor* genus is based on a putatively methanotrophic MAG extracted from a methane-oxidising soil metagenome from Taylor Valley in Antarctica (Genbank accession GCA_002007425.1) [30]. The low number of UBA7966 reference genomes in the GTDB database means, however, that these classifications remain an approximation. The two closest orders to UBA7966 that contain cultured representatives, Beggiatoales and Nitrosococcales, both have members implicated in methanotrophy, sulfur cycling and ammonia oxidation as well as varying degrees of chemolithotrophy and chemoautotrophy [51–54]. On all the contigs assigned to order UBA7966 by CAT, four *pmoCAB* operons were found, with *pmoA* showing 92.9–96.8% identity with *pmoA* from *USCγ-Taylor*. This indicates that, in addition to the methanotrophy of *USCγ-Taylor*, other members of the order UBA7966 could be involved in similar lifestyles.

When analysed with BiG-SCAPE at cut-off 0.7 (Supplementary Table 4), phosphonates (median *d* = 1421), NRPS/NRPS-like (median *d* = 1262) and bacteriocins seemed to form especially conserved GCFs. Other GCFs were shared with other proteobacterial orders. With 96 BGCs, UBA7966 contributed a similar number of BGCs as the established specialised metabolite producing order Burkholderiales (116 BGCs). However, the BiG-SLiCE distances of UBA7966 were higher than Burkholderiales for all but one BGC class, indicating more novel BGCs (Supplementary Fig. 5).

The potential methanotrophy of UBA7966 suggested the potential presence of methanobactins, but no BGCs corresponding to known methanobactins were found in the dataset. On the other hand, an abundance of DUF692-containing BGCs were observed, grouping into three GCFs. DUF692 proteins are a diverse family of proteins with largely unknown functions, although some are known to be involved in methanobactin biosynthesis [55]. The analysis of three related GCFs containing DUF692 domains (including BGCs from UBA7966 and unclassified gammaproteobacterial contigs) showed that FAM_02526 (two BGCs), FAM_02384 (three BGCs) and FAM_02418 (six BGCs) (Fig. 6B, D) all contained a short (circa 240 bp) ORF followed by first a DUF692-domain containing protein, then a DUF2063-domain containing protein. Furthermore, a putative cation antiporter was found upstream of the precursor peptide. The three families differed by the genes surrounding this core cluster (Fig. 6B). The 11 small translated 240 bp ORFs were aligned using Clustal Omega and a HMM search was made in EBI reference proteome database with a cut-off *E*-value of 1E−10. The resulting 290 protein sequences (almost exclusively from Proteobacteria) plus 11 original sequences were aligned using Clustal Omega and a HMM was generated and visualised using skylign.org. The resulting logo showed a low degree of sequence conservation except for a pattern of six conserved cysteines—some followed by glycines—within 40 amino acids towards the N-terminus, and a slightly conserved hydrophobic patch towards the C-terminus (Fig. 6C). This might represent a potential precursor peptide, with the six cysteines marking the potential core peptide.

The UBA7966 order also contained larger BGCs such as four NRPS/NRPS-PKS BGCs with megasynthase genes with a length of more than 20 Kb, the largest cluster possessing 56 Kb of PKS (seven modules) along with NRPS (three modules) genes. This latter BGC also formed a BiG-SCAPE GCF with several MIBiG BGCs which shared the presence of a small peptide moiety followed by several malonyl units.

### Low numbers of BGC found in other underexplored phyla

**Fig. 7:**
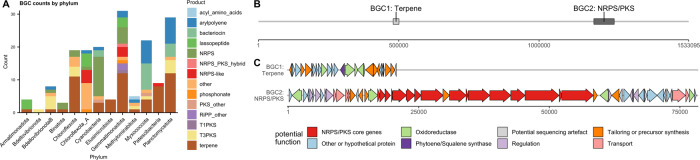
Phylum-level distribution of BGCs in less abundant phyla and BGC map of a Gemmatimonadota contig. **A** Distribution of BGCs among phyla with 31 or fewer BGCs in the dataset. **B** Map of a large Gemmatimonadota contig (order Gemmatimonadales) and BGCs detected on it. **C** Cluster map of proposed functions of genes in BGC1 and BGC2. Functions were predicted from BLASTing against NCBI nr database as well as antiSMASH module predictions. *X* axis represents basepairs. A detailed table of homologous proteins can be found in Supplementary files.

Lower numbers of BGCs were detected in the phyla Gemmatimonadota (31 BGCs), Planctomycetota (29 BGCs), Myxococcota (22 BGCs), Patescibacteria(9 BGCs), Methylomirabilota (5 BGCs), Bdellovibrionota_B (8 BGCs), Elusimicrobiota (4 BGCs), Armatimonadota (4 BGCs) and Binatota (3 BGCs) (Fig. 7A, Supplementary Table 5).

One remarkably long (1.5 Mb, Fig. 7B, C) Gemmatimonadota contig from the order Gemmatimonadales was found to contain two BGCs: one terpene (*d* = 998) and one NRPS/PKS BGC (*d* = 1423). BGC1 contained a phytoene synthase and several related oxidases. BGC2 contained six PKS modules and two NRPS modules as well as modifying enzymes presence of a TonB receptor indicated that the product could serve as a siderophore.

## Discussion

### Metagenomics reveal biosynthetic potential of underexplored bacterial lineages

In our dataset, we found a large number of BGCs in underexplored phyla not usually associated with specialised metabolites. Two previous studies noted NRPS and PKS novelty and diversity in Acidobacteria and Verrucomicrobia [7, 8]. The present study indicates that these underexplored phyla harbour not only novel NRPS/PKS but also new BGCs from many different classes, such as lassopeptides and bacteriocins. While Crits-Christoph et al. [7] highlighted two promising acidobacterial MAGs from the classes Blastocatellia and the Acidobacteriales, in the present sample the classes Blastocatellia and Vicinamibacteria were the main contributors of acidobacterial BGCs. Furthermore, many BGCs were found in other ubiquitous phyla such as Patescibacteria, Gemmatimonadota and Armatimonadota. Three BGCs (two NRPS and one terpene) were placed in the phylum Binatota. The phylum Binatota was proposed by Chuvochina et al. based on a handful of soil MAGs with no cultured representatives [40]. To our knowledge, this is the first description of BGCs belonging to the phylum Binatota. We also discovered highly divergent BGCs from the underexplored Actinobacteriota classes Acidimicrobiia and Thermoleophilia. This suggests that Actinobacteriota, which contain the heavily exploited genus *Streptomyces*, contain unknown lineages harbouring interesting BGC diversity.

In the present dataset, 845 out of 1417 BGCs (59.6%) had a *d* > 900 and 55 (3.9%) had a *d* > 1800 to the closest GCF. These numbers contrast starkly with the 1.2 million original BGCs in the BiG-SLiCE dataset, of which only 13.9 and 0.2% showed *d* > 900 and *d* > 1800, respectively. While it is necessary to note that sequence diversity does not demonstrate chemical diversity, the striking amount of sequence divergence encountered in just one soil sample adds to the mounting evidence that uncultured and underexplored phyla—especially Acidobacteriota—are promising candidates for the discovery of novel specialised metabolites. It is furthermore worth noting that the great biosynthetic diversity found at Mars Oasis is under threat from climate change, with the maritime Antarctic warming by 1–3 °C between the 1950s and the turn of the millennium [56], and, despite a recent pause in this warming trend [57], similar increases in temperature being predicted for later this century as greenhouse gases continue to accumulate in the atmosphere [57, 58].

The large number of terpene BGCs, most of them putatively C30/C40 carotenoids or hopanoids, could be interpreted with respect to the roles of these compounds in membrane function at extreme temperatures [26, 59, 60], as well as UV protection [26, 61]. A previous study similarly noted a high number of pigmented bacteria among isolates from Antarctic samples [26]. Kautsar et al. [47] recorded only 7.8% terpene BGCs in their large-scale survey of publicly available bacterial genomes, as opposed to the ca. 25% in this survey. Previous short-read metagenomic studies of aquatic and soil environments also reported high numbers of terpene BGCs, with terpenes representing between 15 and 50% of the reported BGCs, respectively [62–64]. However, the representativeness of BGC counts obtained through metagenomic studies remains questionable. In this study, for example, the 85.3% abundance-weighted coverage estimated by nonpareil indicates that many less abundant members of the community are not represented in the dataset. Furthermore, small terpene BGCs are easier to assemble than long and repetitive NRPS/PKS BGCs, therefore leading to bias.

In this study, a large number of BGCs were observed in potentially methanotrophic members of the uncultured order UBA7966. Methanotrophic organisms have not usually been linked to specialised metabolite production, except for siderophore-like RiPPs called methanobactins able to scavenge the copper needed for methane and/or ammonia oxygenase enzymes [55]. We reason that the lack of known natural products might be related to difficulties associated with cultivation such as specific nutrient requirements and often slow growth, as well as to the amount of energy, carbon and nitrogen available for specialised metabolite production. While no methanobactin BGCs were seen in UBA7966-classified contigs, examining three gammaproteobacterial DUF692-domain containing GCFs revealed the presence of a potential conserved six cysteine precursor peptide. The conserved cysteines in the potential precursor peptides are resemblant of ranthipeptides (formerly known as SCIFFs), which contain six cysteines in 45 amino acids. Ranthipeptides, however, contain thioethers formed by radical SAM enzymes [65]. DUF692-domain proteins are furthermore known to be involved in methanobactin and TglA-thiaGlu biosynthesis [55, 66], and at least one member has been shown to contain two iron atoms potentially acting as cofactors [66]. All DUF692 protein containing GCFs in the order UBA7966 observed in the present study also contained DUF2063 proteins. DUF2063 family proteins are mostly uncharacterised, though the crystal structure of a member from *Neisseria gonorrhoeae* indicates that DUF2063 might be a DNA-binding domain involved in virulence, and there has been one report of co-occurrence of DUF2063 and DUF692 proteins [67]. Other studies discovered the two neighbouring proteins in operons related to stress response at high calcium concentration [68] in *Pseudomonas* as well as responding to gold and copper ions [69] in *Legionella*. The two genes were also found in the atmospheric methane oxidiser *Methylocapsa gorgona* [70]. We therefore hypothesise that these BGCs could be another form of RiPP involved in chelating metals. While the six cysteines could be involved in forming thioether bonds, disulfide bonds or lanthionine groups like in many other RiPPs, they could potentially also be directly involved in metal coordination as is the case in the group of small metal-binding proteins called metallothioneins [71].

### Long reads make mining and phylogenetic classification of metagenomic BGCs feasible

The advantage of long reads could be observed from comparing the results achieved from long reads vs short reads, with the short reads providing a lower number of BGCs and a significantly lower taxonomic classification success compared to the BGCs assembled and annotated using long reads. While the number of bases used in the assembly was about a third lower for short reads (28 Gb vs 44 Gb), the number of recovered BGCs was more than two-thirds lower (430 BGCs vs 1417 BGCs) and the BGCs assembled from short reads were mostly incomplete. Moreover, this study showed that long-read metagenomes constitute a valuable tool to achieve similar or even improved results to deep short-read metagenomes [7, 62, 63]. For example, Cuadrat et al. used 500 million reads (*c*. 50 Gb if read length was 100 bp) for BGC genome mining of a lake community recovering 243 BGCs with a total of 2200 ORFs, which averages to nine ORFs per BGC indicating small and/or incomplete BGCs [63]. A larger short-read study of microbial mats recovered 1477 BGCs [62]. While this study did not report the number of sequenced bases or BGC completeness, the median BGC length of 103 BGCs from 15 representative and highly complete MAGs was 11.9 Kb, also indicating mostly small and/or incomplete BGCs. Another study by Crits-Christoph et al. [7] used 1.3 Tb of short-read sequence data of grassland soil to mine selected bins of four phyla, recovering a total of 1599 BGCs, 240 of which were NRPS/PKS BGCs, including several large and complete ones [7]. The present study indicates that the long-read approach requires a relatively low sequencing input similar to the two smaller studies to provide a result similar to the larger study. While the contigs, MAGs and BGCs produced using shallow ONT sequencing are not as accurate as the ones produced using deep short-read sequencing, our results show that they can be used to profile the biosynthetic potential of complex environmental samples, estimate their diversity and could be used to guide isolation and heterologous expression strategies. Lower error rates could be achieved through higher coverage in long and short reads as well as advances in long-read basecalling.

We furthermore conclude that contig-level classification using CAT shows advantages compared to genome-resolved metagenomics in single-sample data, where binning is inefficient. Crits-Christoph et al., Chen et al. and Cuadrat et al. used genome-resolved metagenomics [7, 62, 63], in which contigs are binned and bins are mined for BGCs. While it is favourable to attribute BGCs to distinct MAGs, it is viable only when a large number of samples are used, making binning efficient through differential abundance [72]. When using only one sample, binning becomes inefficient and, in our case, missed the vast number of BGCs, with 1139 of 1417 BGCs not being binned. Contig-based classification approaches offer an alternative, but their accuracy is limited by contig length [38] and the classification dependent on the database used. In our data, a contig N50 of >80 Kb provided ample sequence data for accurate classification, leading to >90% classification at phylum level. Usage of GTDB-derived databases ensured improved classification of uncultured taxa, and few conflicts with single-copy core gene-based bin-level classification were detected.

## Conclusions and perspectives

The use of nanopore metagenomic sequencing, binning and contig-based classification approaches using GTDB combined with BGC genome mining allowed us to identify 1417 BGCs, 60% of which were complete, from a wide range of soil bacteria. This confirms and further expands our knowledge of the biosynthetic potential of difficult-to-culture phyla such as Verrucomicrobiota, Acidobacteriota and Gemmatimonadota. In addition, we show that uncultured and underexplored lineages of the well-known producer phyla Actinobacteriota (classes Thermoleophilia and Acidimicrobiia) and Proteobacteria (order UBA7966) show a large biosynthetic potential.

We furthermore demonstrate that ONT long-read sequencing enables the assembly, detection and taxonomic classification of full-length BGCs on large contigs from a highly complex environment using only one sample and 72 Gb sequencing data, which presents a >10-fold reduction compared to studies using short reads to recover large and complete BGCs. While more samples would be needed for improved binning and genome-resolved metagenomics, our approach proved successful in classifying >60% of BGCs at order level.

Even with limited sequencing, we were able to retrieve megabase-sized contigs and one circular genome containing multiple BGCs. With nanopore sequencing becoming more widespread, it will soon be commonplace to profile the biosynthetic potential of uncultured microbes from diverse environments without enormous sequencing efforts. In combination with PCR-based heterologous expression techniques such as DiPaC [12], accessing natural products from metagenomes could be revolutionised, overcoming the need for constructing, maintaining and screening large metagenomic libraries or large sequencing budgets. For remote and endangered environments such as the Antarctic Peninsula, which is warming rapidly due to climate change, these metagenomic strategies will prove especially valuable.

## Supplementary information

Supplemental Figures and Tables

Table of all BGCs with taxonomy, binning, coverage and BiG-SLICE results

BGC genes for figure 4

BGC genes for figure 3

BGC genes for figure 5

BGC genes for figure 7

## Data Availability

The nanopore and Illumina reads generated in this study have been deposited in the Sequence Read Archive with the accession code PRJNA681475.
